# Distinguishing Intermediate and Novice Surgeons by Eye Movements

**DOI:** 10.3389/fpsyg.2020.542752

**Published:** 2020-09-10

**Authors:** Gonca Gokce Menekse Dalveren, Nergiz Ercil Cagiltay

**Affiliations:** ^1^Department of Computer Science, Norwegian University of Science and Technology, Gjøvik, Norway; ^2^Department of Information Systems Engineering, Atılım University, Ankara, Turkey; ^3^Department of Software Engineering, Atilim University, Ankara, Turkey

**Keywords:** eye movement events, eye movement classification, eye tracking, surgical skill assessment, surgical training

## Abstract

Surgical skill-level assessment is key to collecting the required feedback and adapting the educational programs accordingly. Currently, these assessments for the minimal invasive surgery programs are primarily based on subjective methods, and there is no consensus on skill level classifications. One of the most detailed of these classifications categorize skill levels as beginner, novice, intermediate, sub-expert, and expert. To properly integrate skill assessment into minimal invasive surgical education programs and provide skill-based training alternatives, it is necessary to classify the skill levels in as detailed a way as possible and identify the differences between all skill levels in an objective manner. Yet, despite the existence of very encouraging results in the literature, most of the studies have been conducted to better understand the differences between novice and expert surgical skill levels leaving out the other crucial skill levels between them. Additionally, there are very limited studies by considering the eye-movement behaviors of surgical residents. To this end, the present study attempted to distinguish novice- and intermediate-level surgical residents based on their eye movements. The eye-movement data was recorded from 23 volunteer surgical residents while they were performing four computer-based simulated surgical tasks under different hand conditions. The data was analyzed using logistic regression to estimate the skill levels of both groups. The best results of the estimation revealing a 91.3% recognition rate of predicting novice and intermediate surgical residents on one scenario were selected from four under the dominant hand condition. These results show that the eye-movements can be potentially used to identify surgeons with intermediate and novice skills. However, the results also indicate that the order in which the scenarios are provided, and the design of the scenario, the tasks, and their appropriateness with the skill levels of the participants are all critical factors to be considered in improving the estimation ratio, and hence require thorough assessment for future research.

## Introduction

Surgical skill-level assessment is a critical process to ensure competence and prevent clinical errors while developing effective instructional methods for training. It is commonly acknowledged that the skill levels of surgeons can vary depending on numerous elements (Uhrich et al., 2002; Silvennoinen et al., 2009; Chandra et al., 2010; De Blacam et al., 2012). Currently, the assessment of surgical residents’ skill levels is based on subjective approaches and conventional methods, and as such, are prone to bias and questionable rationality (Resnick et al., 1991; Wanzel et al., 2002; Richstone et al., 2010; Meneghetti et al., 2012). Hence, the traditional assessment methods have many shortcomings, such as subjective evaluation, need for expert surgeons and requiring standardized methods of assessment (Moorthy et al., 2003; Ahmidi et al., 2012; Zheng et al., 2015). Several studies have reported that more effective skill-level assessment techniques and training evaluation systems could improve skill-based training, and accordingly patient healthcare (Reiley et al., 2011; D’Angelo et al., 2015; Zheng et al., 2015). Such assessment is also necessary to adapt the content and level of training programs to the surgical residents’ needs and experience, providing sufficient feedback for both trainees and educators, and improving the curriculum (De Blacam et al., 2012; Cundy et al., 2015; Cagiltay et al., 2017; Mizota et al., 2020). On the other hand, simulation environments allow trainees to develop knowledge and necessary skills in a safe and effective environment to apply in real-world applications (Earle, 2006; Van Sickle et al., 2006; Carter et al., 2010; Cristancho et al., 2012; Bilgic et al., 2019). Thus, these virtual and interactive settings in computer-based systems are risk-free (Gallagher and Satava, 2002; Maran and Glavin, 2003; Earle, 2006; Van Sickle et al., 2006; Loukas et al., 2011; Seagull, 2012). Accordingly, several training scenarios have been developed for surgical-skill improvement and assessment requirements for surgery purposes (McNatt and Smith, 2001; Gallagher and Satava, 2002; MacDonald et al., 2003; Jones, 2007; Curtis et al., 2012; Feldman et al., 2012; Hsu et al., 2015). Oropesa et al. (2011) report that adaptation of newly developed tools and techniques to surgical training programs has the possibility to realize an essential role for the assessment of surgical skills. However, there are currently very limited studies considering the precise assessment of these skill levels and they have mainly used the performance metrics for the assessment purposes. For instance, in previous studies, surgeons’ experience levels were assessed for each task in accordance to time, errors, and economy of movement for each hand (McNatt and Smith, 2001; Adrales et al., 2003; Arora et al., 2010; Meneghetti et al., 2012; Perrenot et al., 2012; Mathis et al., 2020). An earlier study objectively estimated the skill levels in a virtual surgical training environment using performance data from surgical residents, such as task completion time, distance, and success (Topalli and Cagiltay, 2019). Another study used electromyography (EMG) activity and muscular discomfort scores to assess the surgical skill levels of surgeons (Uhrich et al., 2002). Oostema et al. (2008) quantified the laparoscopic operative experience for each user using time, path length, and smoothness. Similarly, McDougall et al. (2006) attempted to distinguish surgeons who were laparoscopically naive and those with laparoscopic experience regardless of the degree of previous related surgical experience (McDougall et al., 2006). Oropesa et al. (2013) used motion metrics for assessing laparoscopic psychomotor skills from video analysis.

New technologies offer advantages through recording the eye movements (Atkins et al., 2013; Zheng et al., 2015; Kruger and Doherty, 2016; Gegenfurtner et al., 2017; Jian and Ko, 2017) of surgeons and analyzing the obtained data to provide a cost-effective, automated, and objective basis for assessing their skill levels (Ahmidi et al., 2012; Tien et al., 2014). In this respect, eye-tracking provides objective metrics about human behavior (Yarbus, 1967; Gegenfurtner and Seppänen, 2013; Dogusoy-Taylan and Cagiltay, 2014; Piccardi et al., 2016; Tsai et al., 2016; Bröhl et al., 2017; Jian and Ko, 2017; Tricoche et al., 2020), and these systems have many beneficial properties, making it easy to record and analyze eye-movement data (Gegenfurtner and Seppänen, 2013; Tien et al., 2015; Zheng et al., 2015; Kruger and Doherty, 2016; Gegenfurtner et al., 2017). Thus, eye-tracking is used for assessing and understanding the differences between skill levels in the medical domain (Stuijfzand et al., 2016; Gegenfurtner et al., 2017; McLaughlin et al., 2017; Fichtel et al., 2019). The differences in performances are related to the information-processing capabilities of the left and right hemispheres of the brain, implying that when visual control is required, the dominant hand is likely to perform better than the non-dominant hand and both hands (Hoffmann, 1997). For instance, Vickers (1995) reported the difference in eye movements between expert and novice sports players in foul shooting, in which expert players were seen not to follow the whole shooting process with their eyes but novice players used their eyes to adjust their shots before releasing the ball. In another different though related study, Kasarskis et al. (2001) investigated the differences between the eye movements of expert and novice pilots in a landing simulation and showed that the fixation times of novices were longer than those of the experts because the latter assembled the necessary information more rapidly than the former. Furthermore, the study of Khan et al. (2012) reports significant differences between surgeons from different skill levels on their gaze patterns while watching surgical videos. Another study shows that according to the results of a meta-analysis fixation duration of non-experts is longer than the experts (Gegenfurtner et al., 2011). Understanding these differences, classifying different skill-leveled surgeons appropriately and objectively is another critical task for surgical education programs (McNatt and Smith, 2001; Uhrich et al., 2002; Perrenot et al., 2012; Doleck et al., 2016). By objectively classifying the experience levels of participants, adaptive systems can be developed for their training (Perrenot et al., 2012), and objective standards can be generated (Oostema et al., 2008) as an evaluation criterion for student admission to such programs. However, in the literature, there is no study reporting the precise estimations of the surgeons’ skill levels, especially in relation to their endoscopic surgical skills and eye movement behaviors.

Additionally, there is no consensus on the classification of the skill levels of minimal invasive surgery procedures. Some studies classify it as novice and expert (Aggarwal et al., 2006; Fichtel et al., 2019), where others consider it as novice, intermediate, and expert (Grantcharov et al., 2003; Schreuder et al., 2009; Shetty et al., 2012). Silvennoinen et al. (2009) presented the most detailed classification of the expertise levels of minimally invasive surgery in the following five stages: a beginner who has simply non-specialist knowledge, a novice who has started to develop initial knowledge in the domain, an intermediate who has intensified his/her knowledge, a sub-expert who has general knowledge but incomplete knowledge of a specialized domain, and an expert who has deep knowledge of a specialized domain (Silvennoinen et al., 2009). Earlier studies were mainly conducted with novices and experts for determining their skill levels (Vickers, 1995; Rosser et al., 1998; Kasarskis et al., 2001; Richstone et al., 2010). Even though there have been other efforts to classify the experience levels of surgeons, these were mostly to differentiate novices and experts using a box trainer from their hand movements (Ahmidi et al., 2010; Horeman et al., 2011, 2013; van Empel et al., 2012). Hence, there are very few studies classifying the novice and intermediate endoscopic surgical skill levels based on eye movements with one earlier study (Chmarra et al., 2010) reporting 74.2% accuracy. Although there are several studies referring to simulation based training environments for endoscopic surgery procedures, they are very limited in classifying the endoscopic surgery skill levels in a detail manner and at a higher level of accuracy. Furthermore, there are very limited studies that focus on better understanding the possible contributions of eye-movement analyses on these assessment procedures.

The main aim of this study was to provide insights into novice and intermediate surgical skill level assessment techniques using eye-tacking technology in virtual reality settings. First, it attempts to show a possible relationship between the eye-movements and surgical skill levels for intermediates and novices. Afterward, it attempts to classify these skill levels through their eye-movement behaviors. The purpose behind this initiative was to integrate skill assessment into the curriculum of surgical education programs to provide appropriate feedback and understand the differences between these skill levels. In particular, compared to the gap between novice and expert groups being higher (Silvennoinen et al., 2009), differentiating novice and intermediate skill level groups is particularly challenging. Additionally, to the best of our knowledge, no study has been found that classified these groups based on eye-movement events. For instance, the study of Khan et al. (2012) reports differences on gaze patterns of surgical residents while watching surgical videos. According to the results of this study it was reported that novice surgeons’ eye-gaze patterns generally roam from key areas of the operational field, whereas expert surgeons’ eye-gaze patterns focused on the task-relevant areas (Khan et al., 2012). Also, the study of Gegenfurtner et al. (2011) shows differences on fixation durations between experts and non-experts. According to this study, experts had shorter fixation durations compared to the non-experts (Gegenfurtner et al., 2011). The results of this earlier studies provide evidences indicating differences between novice and intermediate surgical residences’ eye movements while performing surgical tasks. However, these studies consider the differences between experts and non-experts or novices. Hence, earlier studies are very limited considering eye movement behavioral differences of novice and intermediate groups. Therefore, this study was conducted with novices and intermediates and the main hypothesis of the study is that there is a significant difference between intermediate and novice surgical residents’ eye movements considering number of fixations, fixation durations and number of saccades while they are performing surgical tasks under different hand conditions (dominant-hand, non-dominant hand, and both hand). The second hypothesis is that the novice and intermediate surgical residents can be classified by using their eye movements considering number of fixations while they are performing surgical tasks under different hand conditions (dominant-hand, non-dominant hand, and both hand). Hence, the main contribution of this study is classifying the novice- and intermediate-group surgeons through their eye movement events to predict their skill levels accordingly.

For this purpose, in this study, four surgical scenarios were applied and performed in a virtual reality environment under different hand conditions (dominant hand, non-dominant hand, and both hands) using haptic devices controlled by surgical residents. Their eye-movement data was collected while they performed the given tasks in these scenarios. The data was classified into number of fixations, fixation duration and number of saccades events with an open-source eye-movement classification algorithm, Binocular Individual Threshold (BIT) (van der Lans et al., 2011), a velocity-based algorithm for identifying fixations and saccades. BIT is effective since it is a parameter-free fixation-identification mechanism which automatically identifies task- and individual-specific velocity thresholds (van der Lans et al., 2011; Menekse Dalveren and Cagiltay, 2019). BIT classifies fixations and saccades from the data of both eyes based on individual-specific thresholds. This algorithm has advantages over other similar algorithms such as containing binocular viewing and being independent of machine and sampling frequency (van der Lans et al., 2011). Therefore, BIT algorithm can classify the data from varying eye-tracking devices with various sampling frequencies and different level of sensitivities (van der Lans et al., 2011).

## Materials and Methods

In this study, the eye movements of surgical residents were investigated to distinguish their surgical skill levels. Recorded raw eye data was classified and analyzed for differentiating the intermediate and novice surgical residents. This study was carried out in accordance with the protocol which was approved by the Human Research Ethics Board of Atılım University. All subjects gave verbal and written informed consent in accordance with the Declaration of Helsinki.

### Participants

Twenty-three volunteer participants from two departments were recruited for this study: 13 from neurosurgery and 10 from ear-nose-throat (ENT) surgery. Since it is difficult to access surgeons in specialized fields to volunteer for such initiatives, this number of participants is usually considered as acceptable. The majority of the earlier studies were also conducted with limited number of participants, such as 14 surgeons (Wilson et al., 2010), 22 surgeons (Cope et al., 2015), 9 neurosurgeons (Eivazi et al., 2017), 12 students (Uemura et al., 2016), and 19 surgical residents (Hsu et al., 2015). Of the 23 participants in the current study, 14 (three females) were novices with an average age of 27.71 (*SD* = 6.96), with no one having previously performed an endoscopic surgery on their own. On average, the 14 participants had observed 9.57 (*SD* = 13.51) and assisted in 3.57 (*SD* = 10.64) surgical procedures. The remaining nine were intermediates with an average age of 29.33 (*SD* = 1.50) years, who had observed 48.33 (*SD* = 31.62) and assisted in 32.00 (*SD* = 24.19) surgical operations, while performing 16.56 (*SD* = 16.60) operations on average on their own.

### Simulated Scenarios

The simulation scenarios were developed to address the development of required skills for endoscopic skull-base surgery operations which are mainly conducted by ENT and neurosurgery experts. Skills, such as depth perception, 2D and 3D environmental transformations, left-right hand coordination, and hand-eye coordination were aimed to be addressed through the designed scenarios, which were based on surgical experts’ guidance from the neurosurgery and ENT departments of the medical school.

Virtually simulated environments resemble the real world with their visualization and interaction properties (Perrenot et al., 2012; Zhang et al., 2017). In this study, four scenarios were developed based on surgical skill development requirements for endoscopic surgery purposes to collect the participants’ eye movement data.

#### Scenario 1

**FIGURE 1:**
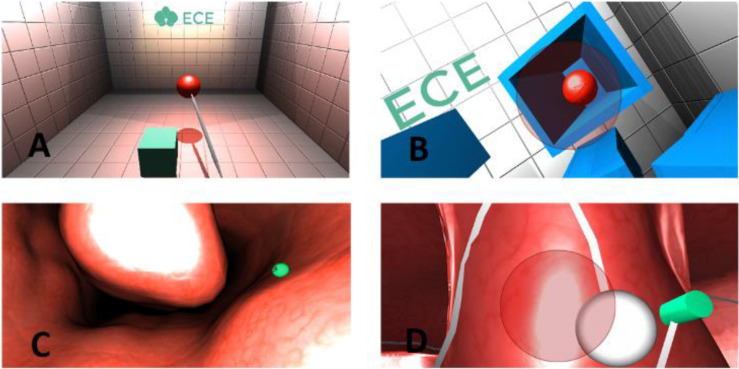
Scenarios [**(A)** scenario 1, **(B)** scenario 2, **(C)** scenario 3, and **(D)** scenario 4].

In this scenario, the participants are expected to use an operational instrument through a haptic device. The scenario is designed to improve depth perception, camera control, 2D-3D conversion, and effective tool usage. With the aid of this tool, the participants have to catch a red ball, which appears randomly in different locations in a room (Figure 1A). The ball must be placed close to the cube; then, the color of the ball turns green. The participants must grab this ball and place it on the green cube. The cube also appears at random positions in the room. This task is repeated 10 times.

#### Scenario 2

A red ball appears randomly in one of the boxes as shown in Figure 1B. The red ball will explode if the approach is at the right angle using the haptic device. This process is repeated 10 times with the aim of developing depth perception and improving the participant’s ability to approach a certain point from the correct angle.

#### Scenario 3

This scenario is a simulated surgical model, in which the participant feels as if they were actually undertaking surgery. As depicted in Figure 1C, the model shows the inside of a human nose. The participants are expected to remove the 10 objects located at different places within the model using the surgical tool. They can move the tool within the model using the haptic device and feel the tissue as the device gives force feedback upon collision with any surface. By using the surgical tool in the most accurate way the participant can complete the operation by carefully removing all the objects from the model.

#### Scenario 4

This scenario is a simulated surgical model and designed like inside of a human nose with similar texture, simulating the field vision of a surgeon during an actual operation. The main task in this scenario is to move an object (the white ball) smoothly on a specified path (Figure 1D). The task is completed when the participant reaches the end of the specified path. The important aspect in this scenario is using the haptic device at a proper angle to keep the object moving on the path.

### Procedure

**FIGURE 2:**
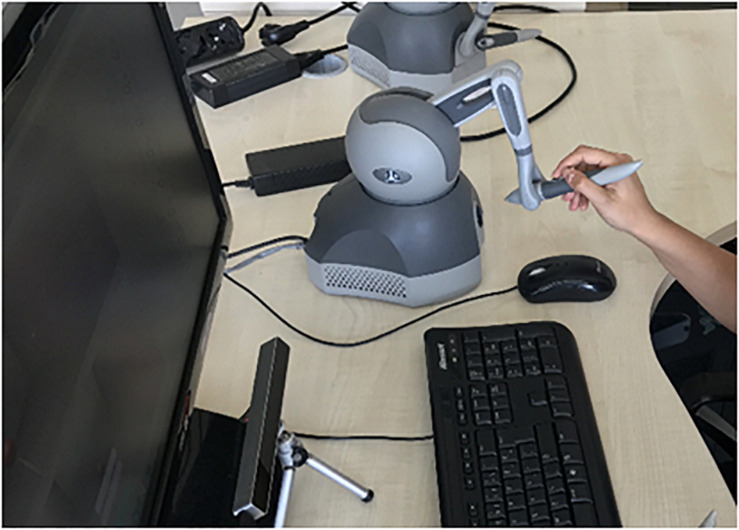
Experiment design.

The participants were informed about the purpose, procedures, and duration of the experiment. The eye-movement data of the surgical residents were collected using an eye-tracking device while they performed the surgical tasks. For the eye data recordings, the Eye Tribe tracker was positioned under the monitor (Figure 2) to provide the information regarding the distance of the user to the monitor and the visibility of the eyes. Therefore, the participants were asked to adjust their seating position to a distance of 70 cm in front of the tracker. The recording frequency was set to 60 Hz and a 9-point calibration process was performed.

**FIGURE 3:**
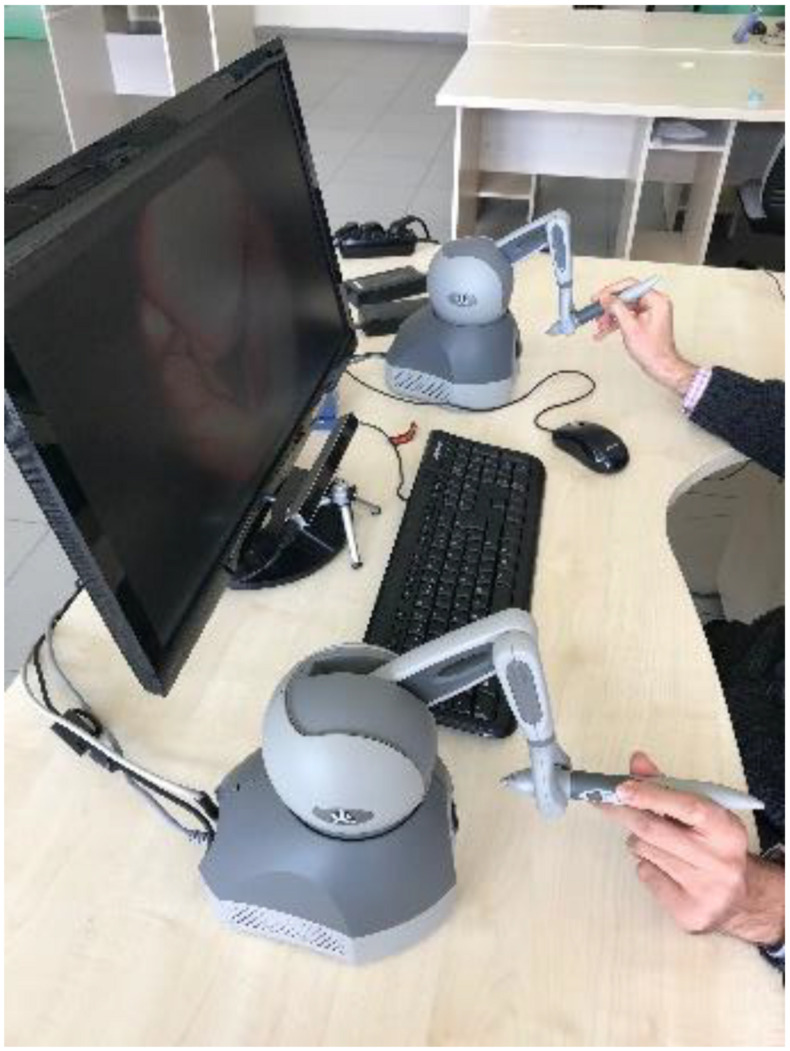
Both-hands condition.

The scenarios were ordered from easy to difficult levels with surgical experts’ guidance from the neurosurgery and ENT departments of the medical school. All the participants performed the scenarios in the same order of 1, 2, 3, and 4 to reduce the chance of negative effects on learning. Each scenario has 10 tasks with a fixed period of time as 20 s. Therefore, each scenario takes 200 s (20 × 10). For added objectivity and reduced order effect, 12 of the participants performed the tasks using their dominant hand, and the remainder with their non-dominant hand. In all cases, the tasks were last performed under the both-hands condition. Each scenario was performed by surgeons using Geomagic Touch haptic devices. All scenarios took place in a simulated environment using a surgical instrument under virtually lit conditions in dominant and non-dominant hand conditions, whereas in the both-hands condition, the participants had to use the light source and the surgical instrument in a coordinated fashion. Therefore, in the both-hands condition, the participants were required to use two haptic devices. They used the first haptic device to hold the surgical instrument with their dominant hand, and they held the second haptic device with their non-dominant hand as a light source to illuminate the operating area as shown in Figure 3. Since the Eye Tribe eye tracker can only record the eye movement data in x and y coordinates, there is a need to classify this raw data into eye movement events, such as fixations; a slow-period eye movement with reduced dispersion and velocity. The classification of raw eye data was performed with BIT, which is an open-source eye movement classification algorithm capable of categorizing raw eye data into fixations and saccades independently from the eye tracker and without specifying the threshold values (van der Lans et al., 2011). In addition, the classified eye movement data was analyzed using statistical methods to predict the skill levels of surgeons based on their eye-movement behaviors.

### Analysis

The analysis of all the data classified by the BIT algorithm was performed using SPSS for the Windows software package (version 23; IBM Corporation, New York, United States) at 95% confidence level. Since the sample size was 23 and the normality assumptions were violated, the non-parametric test technique of Mann-Whitney was used (McCrum-Gardner, 2008) to identify differences between the intermediate and novice surgeons in terms of eye-movement data. This technique is used to compare two independent groups in relation to a quantitative variable (McCrum-Gardner, 2008). A logistic regression analysis was conducted to predict the skill levels of the surgical residents.

## Results

**FIGURE 4:**
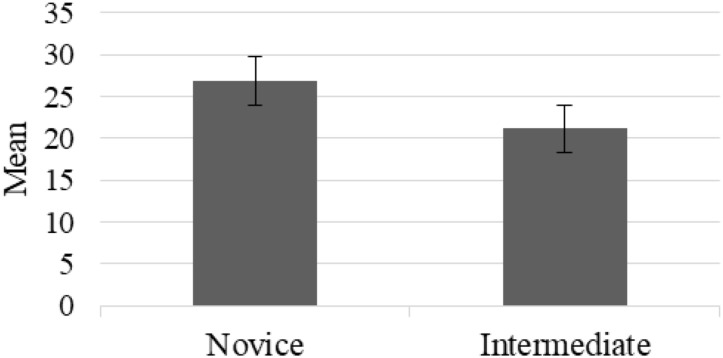
Number of fixations of novice and intermediate surgeons.

**FIGURE 5:**
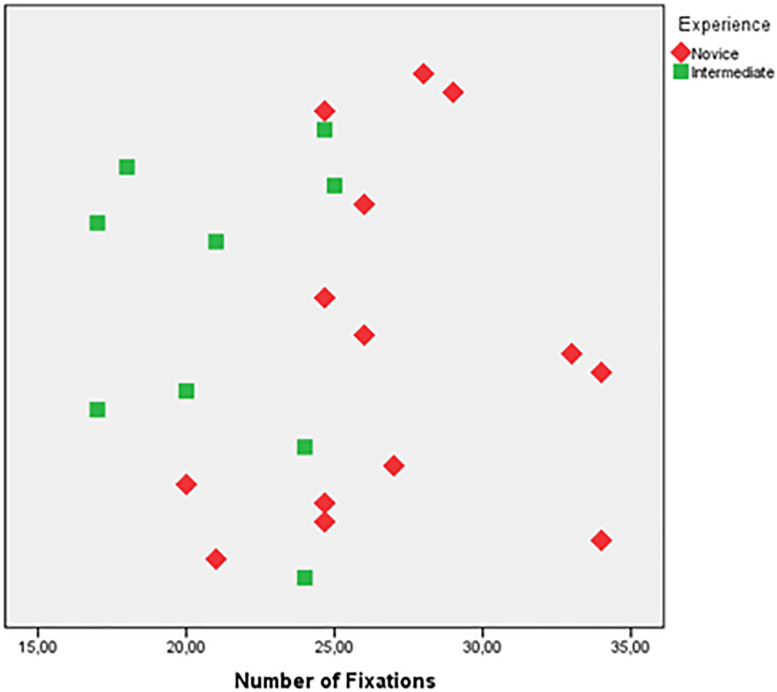
Scatter of the data for number of fixations.

According to the Mann-Whitney test for the novices and intermediates, there was a statistically significant difference in the number of fixations between the two groups (Figure 4) for Scenario 1 under the dominant hand condition. In other scenarios and conditions, no significant difference was found for number of fixations, fixation durations and number of saccades. The scatter plot for number of fixations of Scenario 1 under dominant hand condition was given in Figure 5. In Scenario 1, under the dominant hand condition, the novice surgeons fixated more than the intermediate surgeons according to the results of the BIT algorithm (*U* = 16, *p* < 0.05, *z* = −2.980, *r* = −0.62). This result reveals that there is a potential difference between the novice and intermediate surgical residents with the experience level having an impact on the number of fixations and based on the r value (−0.62) the effect size shows that the difference between intermediate and novice surgeons can be considered as high (Rosenthal et al., 1994). Also, another statistical power analysis was performed to evaluate the effect sizes based on data of this study and the Eta squared (η2) and d Cohen values were found as 0.38 and 1.57, respectively (Lenhard and Lenhard, 2016).

The logistic regression analysis ascertained the effects of the number of fixations, fixation duration and the dominant hand on the prediction of the participants’ skill level. According to the results, in the dominant-hand condition for Scenario 1, the logistic regression model was statistically significant, x2(3) = 15.661, *p* = 0.001. The model explained 66.9% (Negelkerke R2) of the variance in the skill level, and correctly classified 91.3% of all the cases. For Scenario 2, the logistic regression model was not statistically significant, x2(3) = 6.445, *p* = 0.092. The model explained 33.1% (Negelkerke R2) of the variance in the skill level, while accurately classifying 69.6% of the cases. For Scenario 3, the logistic regression model was not statistically significant, x2(3) = 5.449, *p* = 0.142. The model explained 28.6% (Negelkerke R2) of the variance in the skill level with 65.2% of the cases correctly classified. Lastly, in Scenario 4, the model was not statistically significant, x2(3) = 4.941, *p* = 0.176, explaining 26.2% (Negelkerke R2) of the variance in the skill level and correctly classifying 56.5% of the cases.

According to the results, in the non-dominant hand condition for Scenario 1, the logistic regression model was statistically significant, x2(3) = 9.605, *p* = 0.022. The model explained 46.3% (Negelkerke R2) of the variance in the skill level and correctly classified 65.2% of all cases. For Scenario 2, the model was also not statistically significant, x2(3) = 4.830, *p* = 0.185, explaining 25.7% (Negelkerke R2) of the variance in the skill level and correctly classifying 56.5% of the cases. For Scenario 3, the logistic regression model was statistically significant, x2(3) = 10.375, *p* = 0.016, explaining 49.2% (Negelkerke R2) of the variance in the skill level and correctly classifying 73.9% of all cases. For Scenario 4, the model was not statistically significant, x2(3) = 5.388, *p* = 0.146 since it explained 28.3% (Negelkerke R2) of the variance in the skill level and correctly classified 56.5% of the cases.

Finally, in the both-hand condition for Scenario 1 the logistic regression model was statistically significant, x2(3) = 14.289, *p* = 0.003, explaining 62.7% (Negelkerke R2) of the variance in the skill level and correctly classifying 73.9% of all cases. For Scenario 2, the model was not statistically significant, x2(3) = 7.440, *p* = 0.059 as it 37.5% (Negelkerke R2) of the variance and classified 65.2% of the cases. For Scenario 3, the model was statistically significant, x2(3) = 8.670, *p* = 0.034 and explained 42.6% (Negelkerke R2) of the variance in the skill level and correctly classified 65.2% of all cases. For Scenario 4, the logistic regression model was again not statistically significant, x2(3) = 8.031, *p* = 0.45 as it explained 39.9% (Negelkerke R2) of the variance in the skill level and correctly classified 60.9% of the cases.

## Discussion

The results of this study show that it is possible to objectively evaluate the skill levels of novice and intermediate surgeons using eye-movement events, such as number of fixations. This result supports the findings of earlier studies (Gallagher and Satava, 2002; Andreatta et al., 2008; Richstone et al., 2010; Gegenfurtner et al., 2011; Khan et al., 2012). For instance, studies indicating that surgical skill levels can be objectively evaluated by eye-tracking metrics through virtually simulated and live environments (Andreatta et al., 2008; Richstone et al., 2010). Additionally, the findings are in parallel with the results of another earlier study showing that experienced laparoscopic surgeons performed the tasks significantly faster, with fewer errors and more economy in the movement of instruments, and greater consistency in performance (Gallagher and Satava, 2002). Although this is more challenging than comparatively evaluating the skill levels of novice and expert surgeons, this study shows that it is possible to classify intermediate and novice level surgical residents through their eye movement behaviors with a high accuracy. Hence, the main contribution of this current study is showing the eye movement behavioral differences of novice and intermediate surgeons and classifying these groups with a high accuracy (91.3%) which are not reported in the earlier studies. This assessment process could be used to accurately monitor the acquisition of skills throughout training programs at a much earlier stage.

However, the results indicate that in different scenarios, the accuracy of estimations may change (Table 1). Iqbal et al. (2004) reported that the difficulty level of the tasks demanded longer processing times and brought higher subjective ratings of mental workload. In other words, the difficulty level of scenarios needs to be designed in parallel with the skill levels of the trainees. When the tasks in the scenario are too easy or too hard in relation to the trainees’ skill levels, then it could be hard to assess their levels with these tasks. Another study stated that the fidelity level was an important factor affecting mental workload (Munshi et al., 2015). Accordingly, the fidelity levels of the scenarios could be another consideration in designing training scenarios according to a specific skill level. For a better integration of these simulation-based training environments into traditional surgical education programs, further studies need to be conducted to standardize these training scenarios. Therefore, the order of the scenarios and the design of the tasks defined for each scenario may also have an effect on these different findings, necessitating additional research to gain a more thorough understanding of the affective factors on the accuracy of estimations.

**TABLE 1:** Logistic regression results for all conditions and scenarios.

Scenario	Dominant hand		Non-dominant hand		Both hand	
	%	*p*	%	*p*	%	*p*
1	91.3	0.001*	65.2	0.020*	73.9	0.003*
2	69.6	0.092	56.6	0.185	65.2	0.059
3	65.2	0.142	73.9	0.016*	65.2	0.034*
4	56.5	0.176	56.5	0.146	60.9	0.450

*Significant results (p < 0.05).

## Conclusion

The use of eye-movement events is an objective approach which does not require the time and expenditure required to employ expert evaluators. The results of this study reveal that by appropriately designing scenarios addressing both skills under specific conditions (as in the case of the dominant hand condition in Scenario 1 in this study) it may be possible to distinguish the levels of intermediate and novice surgeons through their eye behaviors, up to 91.3%. The highest ratio of the estimation was achieved in Scenario 1 under the dominant hand condition, which implies several possibilities. First, the content and design of the assessment scenarios should be in line with the skill levels of the participants. Second, the design of the scenarios and tasks that require specific skills and their appropriateness with the skill levels of the participants is another critical factor in improving the estimation ratio. In other words, for this group of participants, the design of Scenario 1 was found to be more appropriate for skill assessment purposes. Third, the order of the scenarios implemented in the research procedure is also of significance; for example, as Scenario 1 was implemented first, the accuracy of the assessment regarding the other scenarios might have declined due to the learning affect.

In our earlier study (Topalli and Cagiltay, 2019), the best estimation accuracy was 86% using features, such as time, distance, camera distance, success, catch time, error time, error distance, and deviation count under dominant hand, non-dominant hand and both-hands conditions. This study showed better accuracy when the participants’ eye-movement events were analyzed. Accordingly, this study provides evidence that the eye-movement behaviors of the surgical residents are very important for assessing their skill levels.

Based on the results of this study, eye movements can provide insights into the skill levels of surgical residents and their current education program. In light of this information, it might be beneficial to properly design and organize simulation-based instructional systems for surgical training programs integrating eye movement behavioral analysis into their current methods. Additionally, the progress levels of trainees can be continuously and objectively assessed, and accordingly guidance can be given during training programs.

In conclusion, performance evaluations through computer-based simulation environments by considering the eye movements of surgeons can be improved. In the near future, data obtained from the eye movements of surgical residents can potentially positively support the current evaluation methods by adding to the degree of objectivity. Future studies considering the eye movement data of surgical residents synchronized with their performance data, as well as hand movement data can provide even better results on these estimations and potentially improve the level of prediction. Additionally, by analyzing the performance data (such as task accuracy, task duration and number of errors) in correlation with the eye movements of the trainees’ deeper insights about the possible relationships between eye movements and surgical skill levels can be gained. Furthermore, future studies are required in consideration of the higher level of expertise in the endoscopic surgery field.

## Data Availability Statement

The datasets generated for this study are available on request to the corresponding author.

## Ethics Statement

The studies involving human participants were reviewed and approved by the Ethics Committee of the Atılım University. The participants provided their written informed consent to participate in this study.

## Author Contributions

NC designed the experiment. GM analyzed the data. GM and NC wrote the article. Both authors contributed to the article and approved the submitted version.

## Conflict of Interest

The authors declare that the research was conducted in the absence of any commercial or financial relationships that could be construed as a potential conflict of interest.
